# Prognostic Value of LGR5 in Colorectal Cancer: A Meta-Analysis

**DOI:** 10.1371/journal.pone.0107013

**Published:** 2014-09-05

**Authors:** Qing Chen, Xin Zhang, Wei-Min Li, Yu-Qiang Ji, Hao-Zhe Cao, Pengsheng Zheng

**Affiliations:** 1 Department of Reproductive Medicine, the First Affiliated Hospital, Xi’an Jiaotong University Medical School, Xi’an, the People’s Republic of China; 2 Department of Gynaecology and Obstetrics, the Second Affiliated Hospital, Xi’an Jiaotong University Medical School, Xi’an, the People’s Republic of China; 3 Department of Nutrition, the First Affiliated Hospital, Xi’an Jiaotong University Medical School, Xi’an, the People’s Republic of China; 4 Institute of Cardiovascular research, the First Hospital of Xi’an, Xi’an, the People’s Republic of China; Medical University of Graz, Austria

## Abstract

**Objective:**

Leucine-rich repeat-containing G protein-coupled receptor 5 (LGR5) has recently been reported to be a marker of cancer stem cells (CSCs) in colorectal cancer (CRC), and the prognostic value of LGR5 in CRC has been evaluated in several studies. However, the conclusions remain controversial. In this study, we aimed to evaluate the association between the expression of LGR5 and the outcome of CRC patients by performing a meta-analysis.

**Methods:**

We systematically searched for relevant studies published up to February 2014 using the PubMed, Web of Science, EMBASE and Wangfang databases. Only articles in which LGR5 expression was detected by immunohistochemistry were included. A meta-analysis was performed using STATA 12.0, and pooled hazard ratios (HRs) with 95% confidence intervals (CIs) were used to estimate the strength of the association between LGR5 expression and the prognosis of CRC patients.

**Results:**

A total of 7 studies comprising 1833 CRC patients met the inclusion criteria, including 6 studies comprising 1781 patients for overall survival (OS) and 3 studies comprising 528 patients for disease-free survival (DFS). Our results showed that high LGR5 expression was significantly associated with poor prognosis in terms of OS (HR: 1.87, 95% CI: 1.23–2.84; P = 0.003) and DFS (HR: 2.44, 95% CI: 1.49–3.98; P<0.001). Further subgroup analysis revealed that many factors, including the study region, number of patients, follow-up duration and cutoff value, affected the significance of the association between LGR5 expression and a worse prognosis in patients with CRC. In addition, there was no evidence of publication bias, as suggested by Begg’s and Egger’s tests.

**Conclusions:**

The present meta-analysis indicated that high LGR5 expression was associated with poor prognosis in patients with CRC and that LGR5 is an efficient prognostic factor in CRC.

## Introduction

Colorectal cancer (CRC) is the most common malignancy of the gastrointestinal tract worldwide. As one of the leading causes of cancer-related mortality [1], CRC accounts for more than 600,000 deaths every year [2]. Despite advances in curative surgery and adjuvant therapy, as well as extensive CRC-focused research over the past 20 years, the 5-year survival rate is still poor [3]. Relapse, metastasis and drug resistance are the main factors contributing to the high mortality and poor survival rate of this disease [4]. Increasing evidence suggests that a population of self-renewing tumor cells, known as cancer stem cells (CSCs), is responsible for tumor progression, relapse, metastases and therapeutic resistance [5],[6]. Therefore, the identification of CSCs is crucial in the search for therapeutic targets and useful prognostic markers for CRC.

Becker et al. suggested that leucine-rich repeat-containing G protein-coupled receptor 5 (LGR5) may be a better marker of CSCs in CRC [7]. LGR5 was initially identified as an orphan G protein-coupled receptor (GPCR) that belongs to the subfamily of glycoprotein hormone receptors [8], and it contains a large extracellular domain with 17 leucine-rich repeats and a seven-transmembrane domain. Recently, elevated LGR5 expression has been observed in several types of cancers, including hepatocellular carcinoma [9], CRC [10], ovarian cancer [11], and basal cell carcinoma [12]. In particular, many studies have suggested that LGR5 plays a key role in colorectal carcinogenesis and is associated with the poor outcome of CRC patients [13]–[18]. Although LGR5 allelic variation can affect LGR5 protein expression in colorectal cancers, the somatic LGR5 genotype seems to be relatively stable in primary tumors. Moreover, patients with variant alleles in SNPs of the LGR5 gene showed similar prognosis as patients with wild type LGR5, no significant difference was observed [19]. Therefore, it was expected that LGR5 expression in CRC is an ideal prognostic marker that is correlated with low survival.

In fact, in recent years, many studies have shown that the expression of LGR5 is positively associated with poor prognosis in CRC [13], [15], [17]. However, no correlation was found between the expression of LGR5 and a poor clinical outcome in CRC in another previous study [20]. The prognostic value of LGR5 in CRC patients is controversial, and an insufficient sample size and several other factors likely resulted in the contrary results of different clinical studies. However, to date, there has been no meta-analysis of LGR5 expression and the prognosis of patients with CRC. To clarify the exact prognostic value of LGR5 in CRC, we performed a meta-analysis of eligible studies to investigate the relationship between LGR5 expression and the prognosis of CRC patients.

## Materials and Methods

### Literature search strategy

We searched the PubMed, Web of Science, EMBASE, and Wangfang databases for relevant articles published until March 31st, 2014. The search terms included “LGR5”, “colon cancer”, “rectal cancer”, “colorectal cancer” and “prognosis”. Bibliographies, review articles and pertinent studies were searched manually for additional pertinent studies.

### Selection criteria

The inclusion criteria for the eligibility of a study were as follows: (1) patients with distinctive CRC diagnosis by pathology, (2) an assessment of the relationships between LGR5 expression and the prognosis of CRC patients, (3) sufficient information provided to estimate hazard ratio (HR) for overall survival (OS) or disease-free survival (DFS), and (4) publication with English or Chinese. The following studies were excluded from the analyses: (1) letters, reviews and conference abstracts, due to the limited data, and (2) articles about animal or cell lines. Regarding multiple publications from the same population, only the most recent or the most complete study was included in the analysis.

### Data extraction and quality assessment

Two investigators (Qing Chen and Xin Zhang) reviewed each eligible article independently and extracted information from all the publications meeting the inclusion criteria. The following characteristics were collected from each study: the first author’s name, the year of publication, the country of origin, the number of patients, the age of the patients, the time of follow-up, the disease stage, the cutoff value, LGR5 expression levels and survival data. Disagreements were resolved by discussion until consensus was reached.

Quality assessment was conducted for each of the available studies using the Newcastle-Ottawa quality assessment scale [21]. The score assessed eight items on methodology that were categorized into three dimensions, including selection, comparability and outcome. Interpretation of the scale is performed by awarding points, or “stars”, for high-quality elements. The stars are then added up and used to compare study quality in a quantitative manner. We specified a priori that a score of 7 or higher indicated high quality, a score of 5 or 6 indicated moderate quality, and a score of 4 or less indicated low quality.

### Statistical analysis

Survival outcome data were synthesized using the HR and its 95% confidence interval (CI) to analyze the impact of LGR5 expression on the survival of CRC patients. Several of the included studies provided HRs and 95% CIs, which we pooled directly. Otherwise, we calculated the HR and its 95% CI from available data or a Kaplan-Meier survival curve using Engauge Digitizer version 4.1 (free software downloaded from http://sourceforge.net). By convention, an observed HR>1 implied worse survival for a group with increased LGR5 expression. The impact of LGR5 expression on survival was considered to be statistically significant if there was no overlap of the 95% CI with 1. The heterogeneity among the studies was assessed by a chi-square-based Q statistic test [22], and the I^2^ value was used to quantify the heterogeneity (I^2^ = 0–50%, no or moderate heterogeneity; I^2^>50%, significant heterogeneity) [23]. If homogeneity was not significant (P>0.10 for the Q test), the fixed-effect model was used; otherwise, the random-effect model was used. Publication bias was assessed using Egger’s test and Begg’s test [24], [25]. To adjust for multiple comparisons, we applied the stepdown Bonferroni method, which control for familywise error rate (FEW). In addition, sensitivity analysis was performed to examine the stability of the pooled results. The statistical analyses were conducted using STATA 12.0. All P values were two-sided, and P<0.05 was considered to be statistically significant.

## Results

### Study selection and characteristics

**Figure 1:**
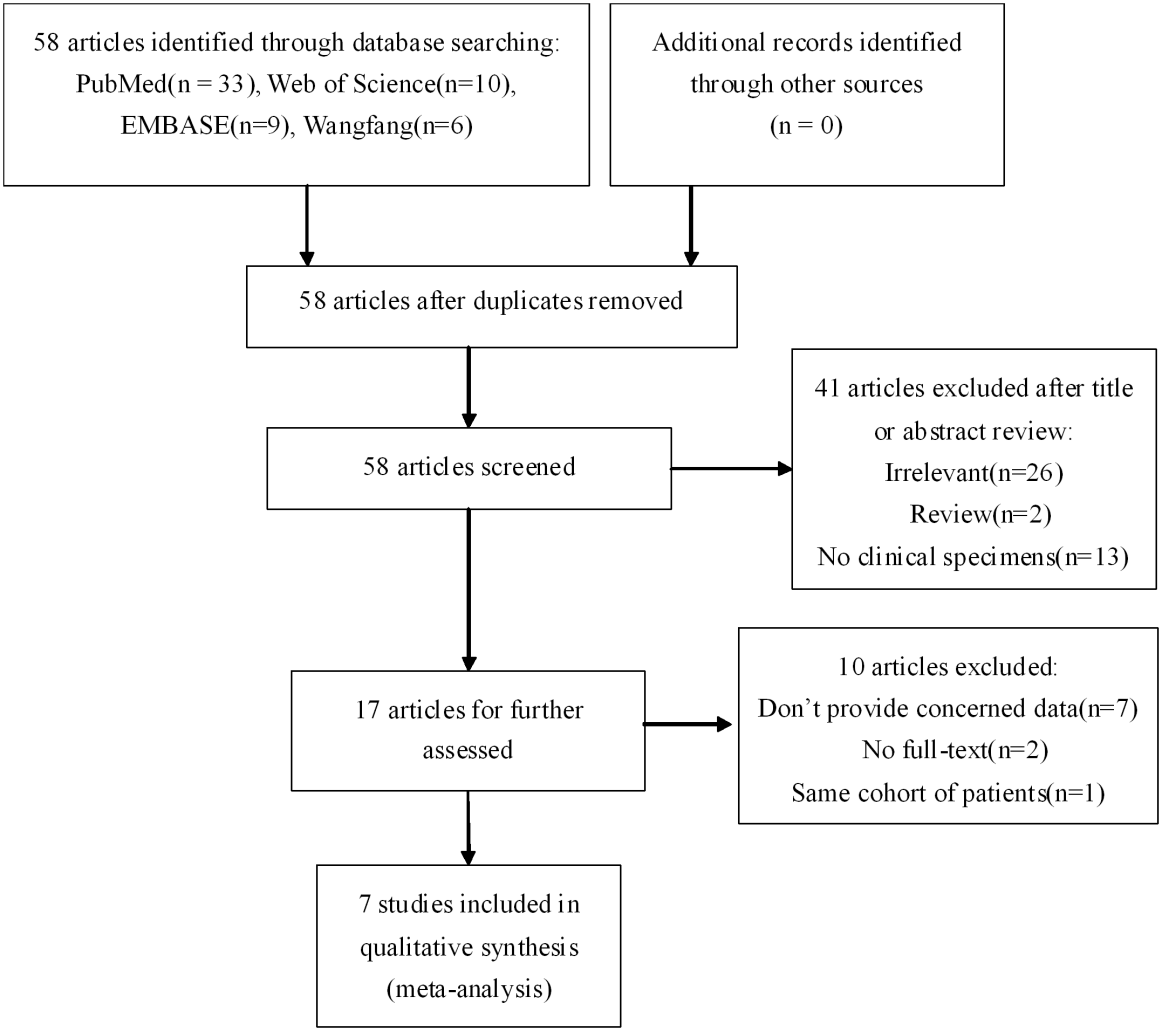
Flow diagram showing the study selection procedure.

As shown in Fig. 1, a total of 58 articles were initially retrieved from the above databases using the search strategy described above, and the details of search results in different databases were shown in Table S1. After reviewing the titles and abstracts of the articles, 41 articles that were irrelevant to our aim were excluded. The remaining 16 articles were then independently scrutinized by two of the authors. Of the articles, 10 articles were excluded: full text could not be found for 2 articles [13], [26], 1 article was about LGR5 expression in the peripheral blood [18], 4 articles did not provide OS or DFS data [10], [27]–[29], 2 articles were about variants or polymorphisms of LGR5[29], [30], and 1 article duplicated a cohort of patients [17]. Eventually, 7 articles met the inclusion criteria and were included in the meta-analysis [14]–[16], .

All 7 eligible articles evaluated the correlation between LGR5 expression in CRC tissues and the prognosis of CRC. The major characteristics of the 7 studies are summarized in Table 1. These studies were published from 2010–2014 and conducted in 3 countries (the USA, Japan and China). The total number of enrolled patients was 1833, with individual samples ranging from 52–891 (median 180). The reported mean age of the patients ranged from 57–66.9 years across the eligible studies. The follow-up period ranged from 28.3–180 months. Several of the studies defined the cutoff value using complex scoring metrics combining the intensity and percentage of LGR5 expression, whereas other studies only used the percentage of LGR5 expression. Positive LGR5 expression was observed to range from 36–60%. There were 2 studies that utilized both OS and DFS to assess the prognostic value of LGR5 expression in CRC patients [16], [32]. Additionally, 4 studies used only OS as an indicator [15], [20], [31], [33], and 1 study used only DFS [17]. HRs and 95% CIs were directly obtained from 5 studies, and for the remaining 2 studies, HRs and 95% CIs were extrapolated from Kaplan-Meier curves. Overall, the studies included in mate-analysis were of high quality, and of the 7 studies, 3 (43%) scored 8, 3 (43%) scored 7 and 1 (14%) scored 6.

**Table 1:** Characteristics of included studies.

First author	Year	Country	Number ofpatients	Age(mean)	Follow-up(mean)	Tumor differentiation(W, M/P)	Stage (I II /III IV)	Cutoff	LGR5(H/L)	Outcomes measuredHR (95% CI)	Study quality#
He [31]	2014	China	53	NA	50 months	34/19	30/23	NA	38/15	OS 2.48 (1.52–4.28)	7
Hsu [16]	2013	China (Taiwan)	296	63.5	28.3 months	262/34	151/145	≥180^a^	121/175	OS 2.33 (1.45–3.86)	8
										DFS 2.46 (1.25–4.83)	
Saigusa [17]	2013	Japan	52	64.5	7 years	44/8	33/19	≥6.62^a^	19/33	DFS 3.94 (1.12–13.87)	7
Ziskin [20]	2012	USA	891	66	15 years	693/160	393/401	>1^a^	332/559	OS 1.09 (0.93–1.28)	6
						Miss data 38	Miss data 97				
Wu [15]	2012	China	192	58.1	59 months	129/63	117/75	>5^a^	108/84	OS 2.77 (1.62–4.73)	8
Takahashi [32]	2011	Japan	180	66.9	2.93 years	166/14	97/83	>8^a^	90/90	OS 1.42 (0.57–3.53)*	8
										DFS 1.91 (0.80–4.52)*	
Peng [33]	2010	China	169	57	100 months	137/32	107/62	≥10%^b^	102/67	OS 1.84 (1.06–3.18)*	7

HR: Hazard ratio; OS: Overall survival; DFS: Disease free survival; H: High expression; L: Low expression; W: Well; M: Moderate; P: Poor; NA: Not available.

a complex scoring,

b percentage;

*estimated by survival curves;

# Study quality was judged based on the Newcastle-Ottawa Scale (range, 1–9).

### LGR5 expression and prognosis of CRC

**Figure 2:**
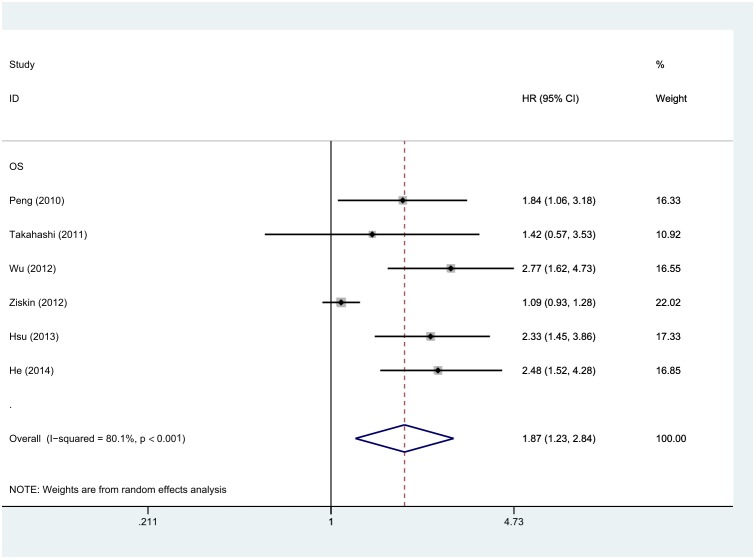
Forest plot of the hazard ratio (HR) for the association of LGR5 expression with overall survival (OS) in colorectal cancer patients in 5 studies. HR>1 implied poor survival, and high LGR5 expression was significantly associated with worse OS in CRC patients.

**Figure 3:**
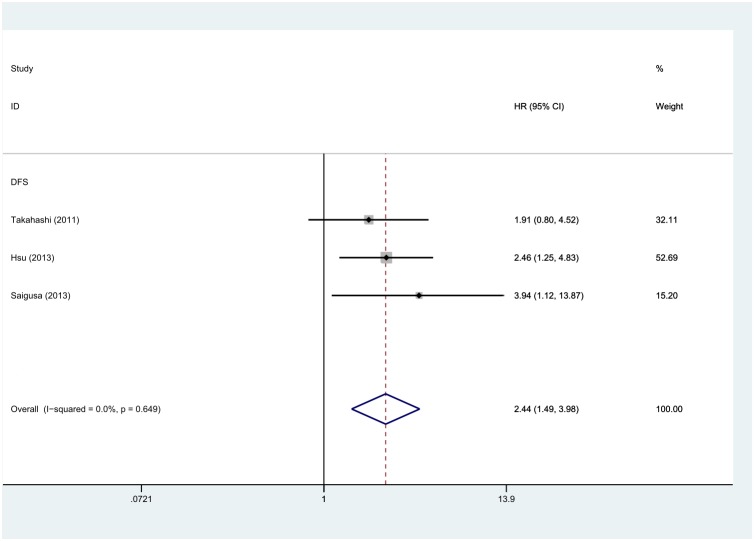
Forest plot of the hazard ratio (HR) for the association of LGR5 expression with disease-free survival (DFS) in colorectal cancer patients in 3 studies. HR>1 implied poor survival, and high LGR5 expression was significantly associated with worse DFS in CRC patients.

6 out of the total of 7 studies reported data on LGR5 expression and OS in CRC [15], [16], [20], [31]–[33], so the combined data from all 6 studies were pooled in the meta-analysis. As shown in Fig. 2, due to a significant degree of heterogeneity (I^2^ = 80.1%, P<0.001), the pooled HRs and 95% CIs were calculated using the random-effect model (REM). The results showed that high LGR5 expression was associated with poor OS in CRC (HR: 1.87, 95% CI: 1.23–2.84, P = 0.003, REM). Additionally, the pooled HR for OS was 1.34 (95% CI: 1.17–1.54, P<0.001) calculated by the fixed-effect model (FEM). 3 of 7 studies reported data on LGR5 expression and DFS, and as shown in Fig. 3, high LGR5 expression was significantly correlated with DFS, with a pooled HR estimate of 2.44 (95% CI: 1.49–3.98, P<0.001, FEM). No significant heterogeneity was observed (I^2^ = 0%, P = 0.649).

We conducted a subgroup analysis of the association of LGR5 expression with OS by study region, patient number, age, follow-up, tumor stage and cutoff value, and the main results are shown in Table 2. The results showed that LGR5 expression was significantly correlated with OS (HR: 2.52, 95% CI: 1.75–2.89) in patients from Asian countries, but not in patients from non-Asian countries (HR: 1.09, 95% CI: 0.93–1.28), here, we should note the fact that only study from non-Asian countries (USA) was included. The subgroup analysis showed a significant correlation between LGR5 expression and OS in studies in which the number of patients was less than 200 (HR: 2.22, 95% CI: 1.66–2.98). The subgroup meta-analysis of the studies with a follow-up period of less than 60 months indicated that high LGR5 expression was a predictor of poor OS (HR: 2.38, 95% CI: 1.79–3.15). When grouped according to the stage of CRC, a significant relationship between LGR5 expression and OS was observed in both the early stage (HR: 2.27, 95% CI: 1.52–3.38) and the later stage (HR: 1.70, 95% CI: 1.01–2.87) of CRC. We also observed a statistically significant effect of LGR5 expression on OS based on the studies using a cutoff >6, with an HR of 2.09 (95% CI: 1.36–3.21). Additionally, the results also showed that the high LGR5 expression was independent predictor of poor OS in CRC patients with the mean age ≤60 (HR: 2.27, 95% CI: 1.52–3.38). Because of the low number of studies, the subgroup analysis of the association of LGR5 expression with DFS was not further pursued.

**Table 2:** Stratified analysis of pooled hazard ratios for colorectal cancer patients with High LGR5expression.

Variable	No. of studies	No. of patients	HR (95% CI)	P value		Heterogeneity		Model used
				P (Z)	P (BON)	I^2^ (%)	P value	
Total OS	6	1781	1.87 (1.23–2.84)	0.003	0.015	80.1%	<0.001	REM
Region								
Asian	5	890	2.25 (1.75–2.89)	<0.001	<0.001	0%	0.694	FEM
No-Asian	1	891	1.09 (0.93–1.28)	0.290	1	-	-	Not applicable
No of patients								
>200	2	1187	1.54 (0.73–3.22)	0.256	0.256	88%	0.004	REM
≤200	4	594	2.22 (1.66–2.98)	<0.001	<0.001	0%	0.532	REM
Age								
>60	3	1367	1.49 (0.85–2.62)	0.159	0.318	76.5%	0.014	REM
≤60	2	361	2.27 (1.52–3.38)	<0.001	<0.001	8%	0.297	REM
Follow up (month)								
>60	2	1060	1.32 (0.81–2.17)	0.269	0.538	68.9%	0.073	REM
≤60	4	721	2.38 (1.79–3.15)	<0.001	<0.001	0%	0.666	REM
Stage (I II /III IV)								
>1.5	2	361	2.27 (1.52–3.38)	<0.001	<0.001	8%	0.297	REM
≤1.5	4	1420	1.70 (1.01–2.87)	0.045	0.090	81.1%	0.001	REM
Cutoff								
>6	2	476	2.09 (1.36–3.21)	0.001	0.004	0%	0.348	REM
≤6	2	1083	1.68 (0.67–4.16)	0.267	0.267	90.6%	0.001	REM

OS: Overall survival; HR: Hazard ratio; CI: Confidence intervals; REM: Random-effect model; FEM: Fixed-effect model.

P (Z): P value for significant test; P (BON): P value from stepdown Bonferroni testing.

### Publication bias analysis

**Figure 4:**
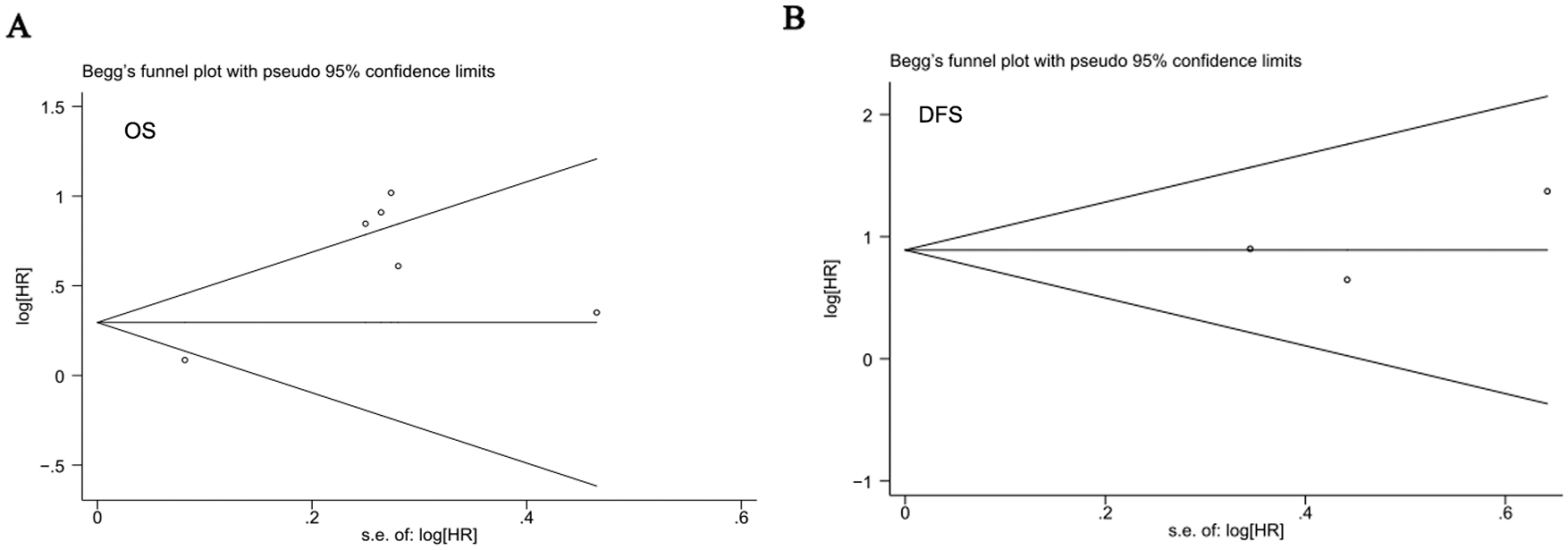
Begg’s funnel plot for all studies included in this meta-analysis. (A) Begg’s funnel plot assessing LGR5 expression and OS in colorectal cancer patients. (B) Begg’s funnel plot assessing LGR5 expression and DFS in colorectal cancer patients.

**Figure 5:**
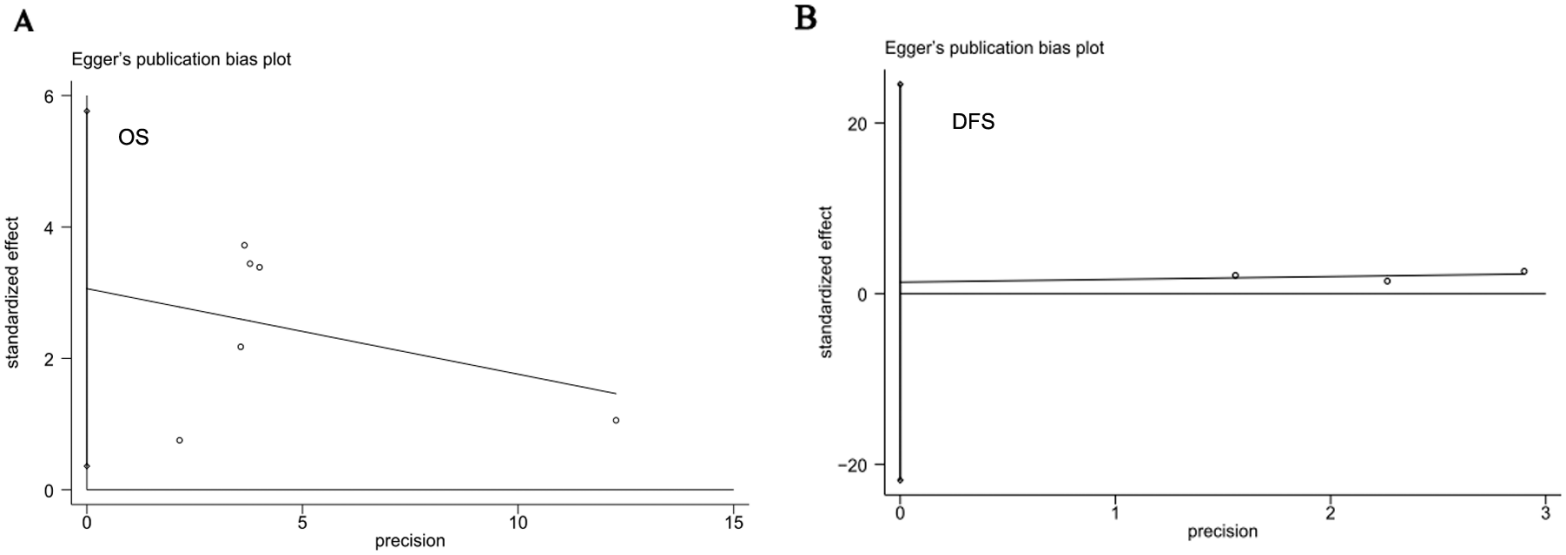
Egger’s test to detect publication bias. (A) Egger’s test assessing LGR5 expression and OS in colorectal cancer patients. (B) Egger’s test assessing LGR5 expression and DFS in colorectal cancer patients.

In this meta-analysis, publication bias was tested using Begg’s funnel plot and Egger’s test. As shown in Figure 4, the funnel plot presented no proof of obvious publication bias in any of the included studies for OS or DFS, and Egger’s test also showed no obvious publication bias in the studies for either of the two outcomes (OS, P = 0.471; DFS, P = 0.749; Fig. 5).

### Sensitivity analysis

To gauge the stability of the result, a sensitivity analysis was conducted, in which one study was deleted at a time. As shown in Table 3, the corresponding pooled HR for OS did not significantly change, regardless of which study was deleted, suggesting that the result was robust. However, the pooled HR for DFS appeared to be significantly altered, which may have been due to the insufficient number of studies.

**Table 3:** Sensitivity analysis for OS and DFS.

Outcome	Study omitted	HR (95% CI)
OS	He (2014) [31]	1.76 (1.12–2.77)
	Hsu (2013) [16]	1.79 (1.12–2.84)
	Ziskin (2012) [20]	2.25 (1.75–2.89)
	Wu (2012) [15]	1.72 (1.12–2.65)
	Takahashi (2011) [32]	1.94 (1.22–3.09)
	Peng (2010) [33]	1.88 (1.15–3.08)
DFS	Hsu (2013) [16]	2.07 (0.28–3.86)
	Saigusa (2013) [17]	2.20 (0.91–3.49)
	Takahashi (2011) [32]	2.57 (0.85–4.30)

## Discussion

CRC is a malignant disease with high mortality worldwide, and its prognosis is still poor, although tremendous progress has been achieved [4]. Recently, alteration of molecular biological markers in tumor tissues has become an important part of predicting the prognosis of patients with malignant tumors. LGR5, known as a target of the Wnt signaling pathway, has been reported to be a potential marker of stem cells in the small intestine and colon [34], [35]. Accumulated studies have shown that LGR5 is closely associated with tumorigenesis and tumor invasion in CRC and is likely to be a relevant marker of CSCs in CRC [12], [36]. CSCs are thought to be responsible for the local invasion, metastasis and recurrence of malignant tumors because of their self-renewal and multi-differentiation potential and thus are also a major obstacle to improving overall cancer survival.

In recent years, the correlation between LGR5 expression and the survival of patients has been explored in many studies due to the key role of LGR5 in tumorigenesis. High LGR5 expression has been extensively reported to be an unfavorable prognostic indicator in various human cancers [13], [15], [33], [37]. However, certain reports have also suggested contrary results for the correlation between LGR5 expression and the prognosis of CRC. For example, Ziskin et al. reported that LGR5 expression was not significantly associated with the outcome of tumors [20] and was not a prognostic marker of CRC (HR: 1.09, 95% CI: 0.93–1.28). To validate the exact relationship between LGR5 expression and the prognosis of patients with CRC, a meta-analysis was performed, including recent related studies and generally using a comprehensive search strategy. To the best of our knowledge, this is the first meta-analysis that has documented the prognostic value of LGR5 in CRC. In the present meta-analysis, through combining the outcomes of 6 published studies, comprising 1781 patients with CRC, we draw the conclusion that high LGR5 expression was significantly associated with poor OS (HR: 1.87, 95% CI: 1.23–2.84, P = 0.003), moreover, the high LGR5 expression was also significantly associated with worse DFS (HR: 2.44, 95% CI: 1.49–3.98, P<0.001) by combining the outcomes of 3 studies comprising 528 patients with CRC. Additionally, the sensitivity analysis showed that the significant association between high LGR5 expression and poor OS was not altered, regardless of whether one of these studies was omitted, suggesting the robustness of this result. However, the significant association between high LGR5 expression and poor DFS was affected by omitting study, which may be due to the small number of studies. In this meta-analysis, the quality assessment was performed independently and reproducibly by two authors according to Newcastle-Ottawa guidelines. By evaluating the articles comprehensively and scientifically, we ensured that the included studies were of high-quality.

Warrants caution is because the baseline characteristics of patients might have affected the conclusion of each included report, including the sample size, follow-up period, clinical stage and cutoff scores for the definition of positive staining, among other aspects. Therefore, further subgroup analysis was performed by study region, patient number, age, follow-up duration, stage and cutoff value. We found that high LGR5 expression was significantly associated with poor OS in the studies performed in Asia (HR: 2.52, 95% CI: 1.75–2.89), but not in one study done outside Asia. Therefore, further investigation is needed to verify whether the prognostic value of LGR5 in CRC is associated with the region inhabited by patients. A significant association was observed between LGR5 expression and poor OS only when the follow-up period was ≤60 months; this finding should be interpreted cautiously due to increased mortality with the prolongation of follow-up time. In addition, the subgroup analysis by clinical stage revealed that high LGR5 expression was significantly associated with poor OS in patients, regardless of the stage (early or late) of CRC, indicating that LGR5 was an independent predictor of prognosis during the progression of CRC. This observation was consistent with the role of LGR5 in carcinogenesis, including enriching CSCs and promoting tumor formation and progression. As there is no uniform standard to define positive LGR5 expression, the cutoff greatly differed among the studies, and the results may also have changed due to the cutoff value. The subgroup analysis showed that LGR5 expression was significantly associated with an unfavorable prognosis in patients with CRC when studies set the cutoff score at >6 (HR: 2.09, 95% CI: 1.36–3.21), suggesting that a higher cutoff score was more likely to lead to a differential conclusion. Previous studies have demonstrated that elevated LGR5 expression significantly correlates with lymphatic invasion, vascular invasion, tumor depth, lymph node metastasis, and tumor recurrence [38], [39].

Publication bias is a major concern in all forms of meta-analysis. In the present study, neither Egger’s test nor Begg’s funnel plot showed evidence of publication bias. However, there are several limitations to the current meta-analysis, as we could not prevent all potential bias among the studies. First, the studies included in our meta-analysis were restricted to only articles published in English or Chinese, and the number was relatively small. Second, heterogeneity among the studies were found in the main analysis, maybe due to the diversity of the techniques used to identify the expression of LGR5. For example, all of the included studies were required to detect LGR5 expression by IHC, but the results of IHC greatly depended on methodological factors such as primary antibody type and antibody concentration, leading to between-study heterogeneity. For this reason, random effect model and subgroup analysis were adopted to adjust for this shortcomings. Third, the method of HR and 95% CI extrapolation should be mentioned, as an HR and a 95% CI calculated from data or extracted from survival curves might be less reliable than those obtained by direct analysis of variance. Additionally, we need to consider the fact that studies with positive results are easily accepted, whereas studies with negative results are often rejected.

In summary, despite the limitations listed above, we found that high LGR5 expression may be an independent risk factor for patients with CRC. Based on currently published articles, high LGR5 expression is an unfavorable prognostic factor in CRC patients and could be helpful to optimize therapeutic schemes. Further, larger prospective studies are needed to validate our results.

## Supporting Information

Table S1 **The search results of relevant articles in different databases.**
(DOC)

Checklist S1 **PRISMA Checklist.**
(DOC)

## References

[1] Jemal A, Bray F (2011) Center MM, Ferlay J, Ward E, et al (2011) Global cancer statistics. CA: a cancer journal for clinicians 61: 69–90. 21296855 10.3322/caac.20107

[2] Ahlquist DA, Zou H, Domanico M, Mahoney DW, Yab TC, et al (2012) Next-generation stool DNA test accurately detects colorectal cancer and large adenomas. Gastroenterology 142: 248–256. 22062357 10.1053/j.gastro.2011.10.031 PMC4017869

[3] Jemal A, Siegel R, Xu J, Ward E (2010) Cancer statistics. CA: a cancer journal for clinicians 60: 277–300. 20610543 10.3322/caac.20073

[4] Kobayashi H, Mochizuki H, Sugihara K, Morita T, Kotake K, et al (2007) Characteristics of recurrence and surveillance tools after curative resection for colorectal cancer: a multicenter study. Surgery 141: 67–75. 17188169 10.1016/j.surg.2006.07.020

[5] Reya T, Morrison SJ, Clarke MF, Weissman IL (2001) Stem cells, cancer, and cancer stem cells. Nature 414: 105–111. 11689955 10.1038/35102167

[6] Visvader JE, Lindeman GJ (2008) Cancer stem cells in solid tumours: accumulating evidence and unresolved questions. Nature Reviews Cancer 8: 755–768. 18784658 10.1038/nrc2499

[7] Becker L, Huang Q, Mashimo H (2008) Immunostaining of Lgr5, an intestinal stem cell marker, in normal and premalignant human gastrointestinal tissue. TheScientificWorldJournal 8: 1168–1176. 10.1100/tsw.2008.148 PMC5848685 19030762

[8] Hsu SY, Liang SG, Hsueh AJ (1998) Characterization of two LGR genes homologous to gonadotropin and thyrotropin receptors with extracellular leucine-rich repeats and a G protein-coupled, seven-transmembrane region. Molecular endocrinology (Baltimore, Md) 12: 1830–1845. 10.1210/mend.12.12.0211 9849958

[9] Yamamoto Y, Sakamoto M, Fujii G, Tsuiji H, Kenetaka K, et al (2003) Overexpression of orphan G-protein-coupled receptor, Gpr49, in human hepatocellular carcinomas with beta-catenin mutations. Hepatology (Baltimore, Md) 37: 528–533. 10.1053/jhep.2003.50029 12601349

[10] Uchida H, Yamazaki K, Fukuma M, Yamada T, Hayashida T, et al (2010) Overexpression of leucine-rich repeat-containing G protein-coupled receptor 5 in colorectal cancer. Cancer science 101: 1731–1737. 20384634 10.1111/j.1349-7006.2010.01571.x PMC11159016

[11] Colon H, Tumors OP, Mcclanahan T, Koseoglu S, Smith K, et al (2006) Identification of Overexpression of Orphan G Protein-Coupled Receptor GPR49 in Human Colon and Ovarian Primary Tumors. Cancer biology & therapy 5: 419–426. 16575208 10.4161/cbt.5.4.2521

[12] Tanese K, Fukuma M, Yamada T, Mori T, Yoshikawa T, et al (2008) G-protein-coupled receptor GPR49 is up-regulated in basal cell carcinoma and promotes cell proliferation and tumor formation. The American journal of pathology 173: 835–843. 18688030 10.2353/ajpath.2008.071091 PMC2527081

[13] Liu Z, Dai W, Jiang L, Cheng Y (2014) Over-expression of LGR5 correlates with poor survival of colon cancer in mice as well as in patients. Neoplasma 61: 177–185. 24063790 10.4149/neo_2014_016

[14] Saigusa S, Inoue Y, Tanaka K, Toiyama Y, Matsushita K, et al (2012) Clinical significance of LGR5 and CD44 expression in locally advanced rectal cancer after preoperative chemoradiotherapy. International journal of oncology 41: 1643–1652. 22923071 10.3892/ijo.2012.1598

[15] Wu XS, Xi HQ, Chen L (2012) Lgr5 is a potential marker of colorectal carcinoma stem cells that correlates with patient survival. World journal of surgical oncology 10: 244. 23153436 10.1186/1477-7819-10-244 PMC3506563

[16] Hsu HC, Liu YS, Tseng KC, Hsu CL, Liang Y, et al (2013) Overexpression of Lgr5 correlates with resistance to 5-FU-based chemotherapy in colorectal cancer. International journal of colorectal disease 28: 1535–1546. 23784051 10.1007/s00384-013-1721-x

[17] Saigusa S, Inoue Y, Tanaka K, Toiyama Y, Kawamura M, et al (2013) Significant correlation between LKB1 and LGR5 gene expression and the association with poor recurrence-free survival in rectal cancer after preoperative chemoradiotherapy. Journal of cancer research and clinical oncology 139: 131–138. 22986809 10.1007/s00432-012-1308-x PMC11824801

[18] Valladares-Ayerbes M, Blanco-Calvo M, Reboredo M, Lorenzo-Patiño MJ, Iglesias-Díaz P, et al (2012) Evaluation of the Adenocarcinoma-Associated Gene AGR2 and the Intestinal Stem Cell Marker LGR5 as Biomarkers in Colorectal Cancer. International journal of molecular sciences 13: 4367–4387. 22605983 10.3390/ijms13044367 PMC3344219

[19] Kleist B, Xu L, Kersten C, Seel V, Li G, et al (2012) Single nucleotide polymorphisms of the adult intestinal stem cell marker Lgr5 in primary and metastatic colorectal cancer. American journal of translational research 4: 279–290. 22937206 PMC3426388

[20] Ziskin JL, Dunlap D, Yaylaoglu M, Fodor IK, Forrest WF, et al.. (2012) In situ validation of an intestinal stem cell signature in colorectal cancer. Gut. 10.1136/gutjnl-2011-301195 22637696

[21] Stang A (2010) Critical evaluation of the Newcastle-Ottawa scale for the assessment of the quality of nonrandomized studies in meta-analyses. European Journal of Epidemiology 25: 603–605. 20652370 10.1007/s10654-010-9491-z

[22] Handoll HH (2006) Systematic reviews on rehabilitation interventions. Archives of physical medicine and rehabilitation 87: 875. 16731227 10.1016/j.apmr.2006.04.006

[23] Higgins JP, Thompson SG, Deeks JJ, Altman DG (2003) Measuring inconsistency in meta-analyses. BMJ: British Medical Journal 327: 557. 12958120 10.1136/bmj.327.7414.557 PMC192859

[24] Egger M, Smith GD, Schneider M, Minder C (1997) Bias in meta-analysis detected by a simple, graphical test. Bmj 315: 629–634. 9310563 10.1136/bmj.315.7109.629 PMC2127453

[25] Begg CB, Mazumdar M (1994) Operating characteristics of a rank correlation test for publication bias. Biometrics: 1088–1101. 7786990

[26] Pitule P, Vycital O, Bruha J, Novak P, Hosek P, et al (2013) Differential expression and prognostic role of selected genes in colorectal cancer patients. Anticancer research 33: 4855–4865. 24222123

[27] Fan X-S, Wu H-Y, Yu H-P, Zhou Q, Zhang Y-F, et al (2010) Expression of Lgr5 in human colorectal carcinogenesis and its potential correlation with beta-catenin. International journal of colorectal disease 25: 583–590. 20195621 10.1007/s00384-010-0903-z

[28] Kleist B, Xu L, Li G, Kersten C (2011) Expression of the adult intestinal stem cell marker Lgr5 in the metastatic cascade of colorectal cancer. International journal of clinical and experimental pathology 4: 327–335. 21577318 PMC3093057

[29] Chai N, Zhang W, Wang Y, Zhou Z, Zhang Y, et al (2013) Lgr5 and CD44 expressions in different types of intestinal polyps and colorectal cancer. Nan fang yi ke da xue xue bao = Journal of Southern Medical University 33: 972–976. 23895835

[30] Gerger A, Zhang W, Yang D, Bohanes P, Ning Y, et al (2011) Common cancer stem cell gene variants predict colon cancer recurrence. Clinical cancer research: an official journal of the American Association for Cancer Research 17: 6934–6943. 21918173 10.1158/1078-0432.CCR-11-1180

[31] He S, Zhou H, Zhu X, Hu S, Fei M, et al. (2014) Expression of Lgr5, a marker of intestinal stem cells, in colorectal cancer and its clinicopathological significance. Biomedicine & pharmacotherapy. http://dx.doi.org/10.1016/j.biopha.2014.03.016 [pii: S0753-3322(14)00044-4, Epub ahead of print]. 10.1016/j.biopha.2014.03.016 24751002

[32] Takahashi H, Ishii H, Nishida N, Takemasa I, Mizushima T, et al (2011) Significance of Lgr5 (+ve) cancer stem cells in the colon and rectum. Annals of surgical oncology 18: 1166–1174. 21125339 10.1245/s10434-010-1373-9

[33] Peng L, Liang C, Liang Y, Hu JJ, Wang J, et al (2010) Higher expression of Lgr5 predicts poor prognosis in human colorectal cancer. Modern Digestion & Intervention 15: 284–287.

[34] Barker N, van Es JH, Kuipers J, Kujala P, van den Born M, et al (2007) Identification of stem cells in small intestine and colon by marker gene Lgr5. Nature 449: 1003–1007. 17934449 10.1038/nature06196

[35] Haegebarth A, Clevers H (2009) Wnt signaling, lgr5, and stem cells in the intestine and skin. The American journal of pathology 174: 715–721. 19197002 10.2353/ajpath.2009.080758 PMC2665733

[36] Merlos-Suárez A, Barriga FM, Jung P, Iglesias M, Céspedes MV, et al (2011) The intestinal stem cell signature identifies colorectal cancer stem cells and predicts disease relapse. Cell stem cell 8: 511–524. 21419747 10.1016/j.stem.2011.02.020

[37] Nakata S, Campos B, Bageritz J, Bermejo JL, Becker N, et al (2013) LGR5 is a marker of poor prognosis in glioblastoma and is required for survival of brain cancer stem-like cells. Brain pathology 23: 60–72. 22805276 10.1111/j.1750-3639.2012.00618.x PMC8028985

[38] Yamanoi K, Fukuma M, Uchida H, Kushima R, Yamazaki K, et al (2013) Overexpression of leucine-rich repeat-containing G protein-coupled receptor 5 in gastric cancer. Pathology international 63: 13–19. 23356221 10.1111/pin.12013

[39] Uchida H, Yamazaki K, Fukuma M, Yamada T, Hayashida T, et al (2010) Overexpression of leucine-rich repeat-containing G protein-coupled receptor 5 in colorectal cancer. Cancer science 101: 1731–1737. 20384634 10.1111/j.1349-7006.2010.01571.x PMC11159016

