# Supplementary material for: Prognostic Value of LGR5 in Colorectal Cancer: A Meta-Analysis
Source: PLoS One. 2014 Sep 5;9(9):e107013. doi: 10.1371/journal.pone.0107013 (PMC4156381; doi:10.1371/journal.pone.0107013)
Supplement: Table S1 — The search results of relevant articles in different databases. (DOC) [file pone.0107013.s001.doc]

Table S1 The search results of relevant articles in different databases

| Database (31.03.2014) | No. | Searches | Results |
| --- | --- | --- | --- |
| Pubmed | 1# | “LGR5” | 380 |
|  | 2# | “LGR5” AND “colon cancer” | 48 |
|  | 3# | “LGR5” AND “rectal cancer” | 5 |
|  | 4# | “LGR5” AND “colorectal cancer” | 64 |
|  | 5# | “LGR5” AND “colorectal cancer” AND prognosis | 8 |
|  | Total | Initial screening by title from 1#, 2#, 3#, 4#, 5# | 33 |
| Web of science | 1# | “LGR5” | 532 |
|  | 2# | “LGR5” AND “colon cancer” | 147 |
|  | 3# | “LGR5” AND “rectal cancer” | 6 |
|  | 4# | “LGR5” AND “colorectal cancer” | 131 |
|  | 5# | “LGR5” AND “colorectal cancer” AND prognosis | 10 |
|  | Total | Initial screening by title from 1#, 2#, 3#, 4#, 5# | 10 |
| Embase | 1# | “LGR5” | 123 |
|  | 2# | “LGR5” AND “colon cancer” | 15 |
|  | 3# | “LGR5” AND “rectal cancer” | 0 |
|  | 4# | “LGR5” AND “colorectal cancer” | 15 |
|  | 5# | “LGR5” AND “colorectal cancer” AND prognosis | 2 |
|  | Total | Initial screening by title from 1#, 2#, 3#, 4#, 5# | 9 |
| Wanfang | 1# | “LGR5” | 107 |
|  | 2# | “LGR5” AND “colon cancer” | 8 |
|  | 3# | “LGR5” AND “rectal cancer” | 2 |
|  | 4# | “LGR5” AND “colorectal cancer” | 6 |
|  | 5# | “LGR5” AND “colorectal cancer” AND prognosis | 1 |
|  | Total | Initial screening by title from 1#, 2#, 3#, 4#, 5# | 6 |
